# Clinical observation of two bone cement distribution modes of percutaneous vertebroplasty in the treatment of thoracolumbar Kümmell’s disease

**DOI:** 10.1186/s13018-020-01774-8

**Published:** 2020-07-09

**Authors:** Ji-Bin Chen, Ya-Ping Xiao, Dong Chen, Jian-Zhong Chang, Te Li

**Affiliations:** 1 Department of Orthopedics, Wuhan Hanyang Hospital, Wuhan University of Science and Technology, Wuhan, 430050 China; 2 Department of Orthopedic Surgery, CR & WISCO General Hospital, Wuhan University of Science and Technology, Wuhan, China; 3 Department of Orthopedic Surgery, General Hospital of Central Theater Command, Wuhan, China

**Keywords:** Kümmell’s disease, Osteoporosis, Vertebral compression fracture, Percutaneous vertebroplasty

## Abstract

**Background:**

In recent years, percutaneous vertebroplasty (PVP) has provided a new option for the treatment of Kümmell’s disease (KD). This retrospective study aimed to investigate the differences in clinical characteristics, clinical efficacy, and related complications between two types of bone cement distribution patterns in the PVP treatment of KD.

**Methods:**

A total of 63 patients with KD from January 2016 to February 2018 who received PVP treatment were examined at least 24 months. According to X-ray distribution modes of bone cement after PVP treatment, they were divided into 2 groups: blocky group (30 cases) and spongy group (33 cases). Clinical features and disease severity preoperatively, and clinical efficacy and related complications postoperatively were statistically compared between the two groups.

**Results:**

The two groups were followed for at least 24 months. The duration of disease, age, Cobb angle, and vertebral compression rate preoperatively were significantly higher in the blocky group than in the spongy group (*P* < 0.05, respectively). The height of vertebral anterior margin and BMD were significantly lower in the blocky group than in the spongy group (*P* < 0.05, respectively). The amount of bone cement injected was significantly greater in the blocky group than in the spongy group (*P* = 0.000). VAS and ODI of the two groups were significantly reduced at the first day, the first year, and the last follow-up postoperatively (all *P* = 0.000) and were maintained at the last follow-up. VAS and ODI postoperatively decreased significantly in the spongy group compared with the blocky group (*P* = 0.000). The correction degrees of kyphosis and vertebral compression postoperatively in the two groups were significantly corrected, but gradually decreased over time (*P* < 0.05), and these correction degrees were significantly higher in the blocky group than in the spongy group, and the postoperative losses were also more serious.

**Conclusions:**

The disease was more serious in the blocky group than in the spongy group. The amount of bone cement, correction degrees of postoperative kyphosis and vertebral compression were significantly higher in the blocky group than in the spongy group, but its postoperative losses of the correction degrees of kyphosis and vertebral compression were also more serious. However, for pain relief and functional recovery, the spongy group was superior to the blocky group. Therefore, the spongy distribution pattern should be formed during the injection of bone cement to obtain better therapeutic effect.

## Background

Kümmell’s disease (KD) is also called avascular necrosis after osteoporotic vertebral compression fractures (OVCFs), post-traumatic vertebral osteonecrosis, vertebral pseudarthrosis, delayed vertebral collapse etc. [1], which is a rare special type of OVCFs [2]. The incidence of KD in OVCFs was reported to be as high as 12.1–42.4% [3, 4]. The thoracolumbar vertebra is the most common site of occurrence. The main clinical manifestations are lower back pain and kyphosis [1]. Imaging examination shows the late-onset vertebral collapse and characteristic change of intravertebral vacuum cleft (IVC) or intravertebral vacuum phenomenon (IVP) [5]. IVP often appear as intravertebral radiolucent shadows that are typically band like or linear in shape and are often accompanied by peripheral sclerosis. MRI imaging can find limited fluid filling in the vertebral body [6].

Percutaneous vertebroplasty (PVP) infuses bone cement into the vertebral body in a minimally invasive manner to stabilize the fractured vertebra, provide early relief of lower back pain, restore partial height of the vertebra, and correct kyphosis [7, 8]. PVP treatment can reduce the risk of wedge shape and nerve damage in patients with KD, so early surgical treatment is recommended [9]. According to the different distribution patterns of vertebral bone cement after PVP treatment for KD, it can be divided into two types of bone cement distribution patterns: blocky type and spongy type [10]. The clinical characteristics of different distribution patterns and whether they produce different clinical effects have not been reported.

Therefore, the author used a retrospective method to analyze the patients with single-segment thoracolumbar KD who treated with PVP surgery in our hospitals from January 2016 to February 2018. The clinical characteristics, clinical efficacy, and related complications of the two types of distribution patterns of bone cement were compared to provide a reference for clinical practice.

## Methods

### Selection criteria

This study was a controlled and retrospective study. The patients were blinded, but the researchers were not completely blinded. Inclusion criteria were as follows: (1) patients with single-segment thoracolumbar KD without new OVCFs in other vertebra; (2) computed tomography (CT) or magnetic resonance imaging (MRI) examination confirmed the existence of IVC. IVC refers to a significant radiolucency (gas containing), which is located centrally or adjacent to the vertebral endplates as seen on CT or plain radiographs. On MRI, according to whether the liquid or gas is full of cracks, the T1-weighted image usually shows low signal intensity, while the T2-weighted image shows high or low signal [11]. (3) The “pain vertebra” located in the physical examination was consistent with the imaging examination. (4) Double-energy X-ray determination of bone mineral density (BMD) *T* value < − 2.5; (5) bilateral pedicle PVP treatment; (6) follow-up time more than 2 years without fall and other trauma. The exclusion criteria were as follows: (1) patients had severe cardiopulmonary dysfunction, coagulation dysfunction, and other intolerance to surgery; (2) local or systemic infection affected the operation; (3) patients had compression symptoms of nerve root or spinal cord; (5) new OVCFs or KD was found in other vertebral bodies. (6) Obvious compression deformation was found in adjacent vertebra.

### General information

**Fig. 1:**
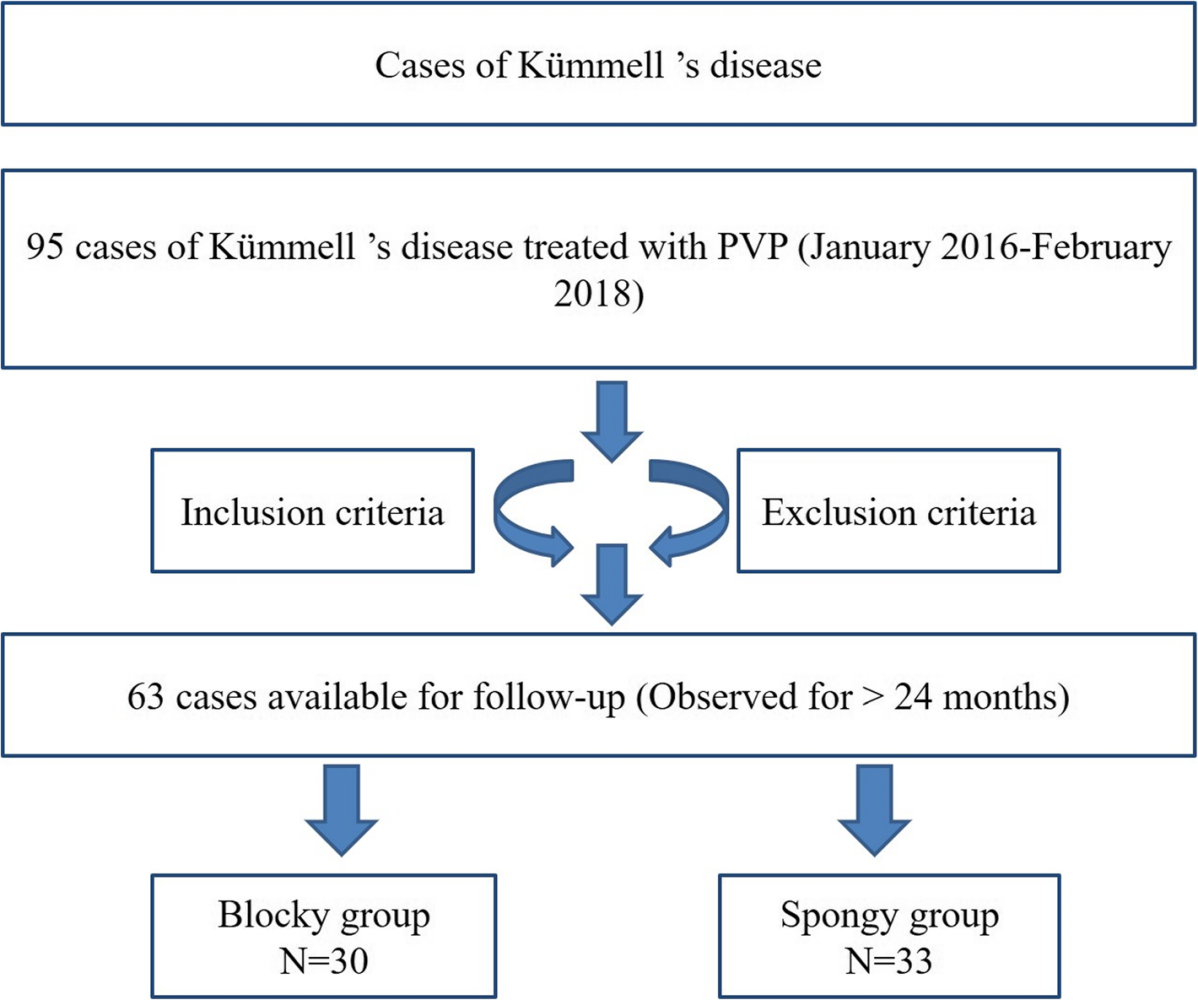
Cases of Kümmell’s disease and follow-up period

**Fig. 2:**
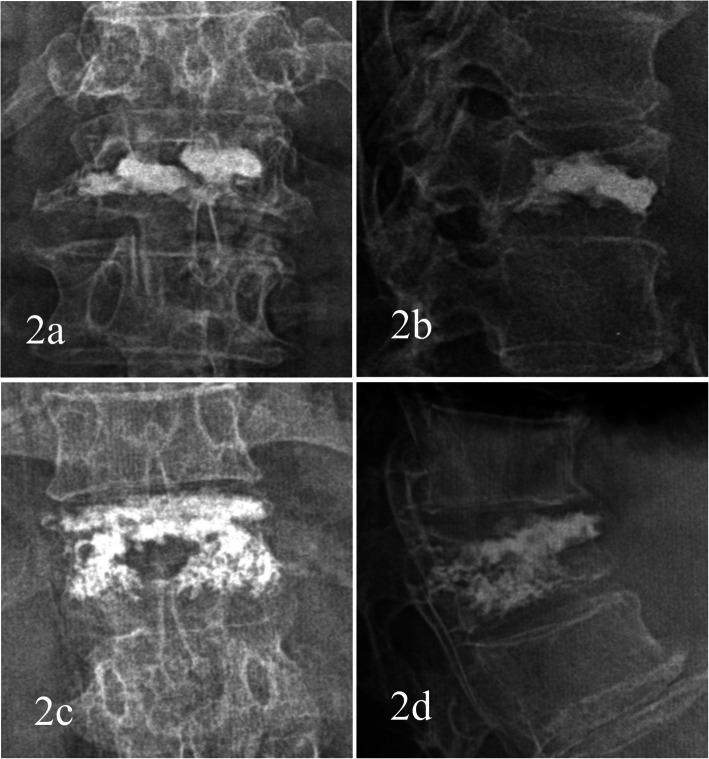
Distribution characteristics of bone cement. **a** Anteroposterior X-ray film of local solid distribution pattern in the blocky group. **b** Lateral X-ray film of local solid distribution pattern in the blocky group. **c** Anteroposterior X-ray film of diffuse distribution pattern in the spongy group. **d** Lateral X-ray film of diffuse distribution pattern in the spongy group

From January 2016 to February 2018, 63 of the 95 patients with KD met the selection criteria and were included in the study, all of whom received PVP treatment (Fig. 1). According to the distribution difference of vertebral bone cement after PVP treatment in the X ray imaging, 63 patients were divided into blocky group (30 cases) and spongy group (33 cases) (Fig. 2). The study was approved by ethics committees of the investigator’s hospitals. Written informed consent of clinical data and images was obtained from all patients.

There were no significant differences in gender, damaged segments, visual analog scale (VAS), and Oswestry Disability Index (ODI) preoperatively between the two groups (*P* > 0.05, Tables 1 and 2). The duration, age, Cobb angle, and vertebral compression rate of the blocky group were significantly higher than these of the spongy group (*P* < 0.05, Table 1). The height of anterior vertebral margin and BMD in the blocky group was significantly lower than those in the spongy group (*P* < 0.05, respectively, Table 1).

**Table 1:** Baseline data of the two groups

Parameters	Blocky group	Spongy group	*t/χ*^*2*^	*P*
Cases	30	33		
Gender			0.057	0.811
Male (cases)	9	9		
Female (cases)	21	24		
Age (years)	76.7 ± 5.60	73.1 ± 6.79	19.587	0.000
Course of disease (months)	5.2 ± 2.34	3.2 ± 1.35	7.834	0.000
Injured vertebral segment			1.271	0.736
T11(cases)	5	6		
T12(cases)	10	13		
L1(cases)	12	9		
L2(cases)	3	5		
BMD (*T* value)	− 4.1 ± 0.91	− 3.9 ± 0.69	2.208	0.028
Anterior vertebral height (mm)	11.0 ± 4.19	11.7 ± 3.80	− 2.292	0.022
Cobb angle (°)	13.2 ± 4.78	12.5 ± 4.08	2.199	0.028
Vertebral compression rate(%)	35.4 ± 8.99	34.2 ± 8.84	2.953	0.003

**Table 2:** Comparison of clinical outcomes between the two groups

Parameters	Blocky group	Spongy group	*t/χ*^*2*^	*P*
Follow-up time(months)	37.6 ± 6.59	37.6 ± 6.86	− 0.077	0.939
Amount of bone cement injected(ml)	4.7 ± 1.55	4.0 ± 1.16	3.897	0.000
Operation time (min)	38.9 ± 5.52	38.7 ± 5.05	0.864	0.388
Intraoperative blood loss (ml)	20.8 ± 2.94	20.7 ± 3.39	0.438	0.661
Fluoroscopy times	18.1 ± 2.85	18.1 ± 3.31	− 0.237	0.813
Bone cement leakage(%)	16.67	21.21	0.211	0.646
Yes (cases)	5	7		
No (cases)	25	26		
VAS				
Before surgery	8.2 ± 1.43	7.9 ± 1.72	1.646	0.100
On the first day after surgery	3.1 ± 0.92*	2.4 ± 0.66*	5.401	0.000
At the first year after surgery	3.5 ± 0.88*	2.6 ± 0.82*	6.508	0.000
At the last follow-up	3.6 ± 0.88*	2.8 ± 0.80*	7.031	0.000
ODI				
Before surgery	72.6 ± 10.89	70.5 ± 10.03	5.293	0.162
On the first day after surgery	29.4 ± 5.42*	24.4 ± 5.13*	24.533	0.000
At the first year after surgery	29.8 ± 5.39*	24.9 ± 5.01*	25.407	0.000
At the last follow-up	29.9 ± 5.41*	25.0 ± 5.56*	22.872	0.000
Adjacent vertebral fractures(%)	16.67	9.09	0.814	0.367
Yes (cases)	5	3		
No (cases)	25	30		
Correction degree of vertebral compression				
On the first day after operation	25.7 ± 4.28	20.4 ± 4.70	21.962	0.000
At the first year after operation	22.8 ± 4.92#	19.6 ± 3.63	13.281	0.000
At the last follow-up	21.5 ± 4.29&*@*	18.9 ± 3.47#	11.952	0.000
Correction degree of kyphosis				
On the first day after operation	26.3 ± 5.34	21.6 ± 5.69	22.467	0.000
At the first year after operation	23.4 ± 5.24#	20.1 ± 5.26#	17.093	0.000
At the last follow-up	21.5 ± 5.17&*@*	19.3 ± 5.19&	14.509	0.001

*Compared to before surgery, *P* = 0.000

^#^Compared to the first day after operation, *P < 0.05*

^&^Compared to the first day after operation, *P = 0.000*

^*@*^Compared to the first year after operation, *P < 0.05*

### Surgical techniques

All operations were performed by the same group of physicians. The patients lied prone to reposition the spine by extending it. Lidocaine was used for local anesthesia. The injured vertebra was located under C-arm X-ray fluoroscopy. The tip of working trocar needle was punctured to the anterior 1/3 of the vertebral body or adjacent to the IVC area through bilateral pedicle approach. Polymethylmethacrylate bone cement (Tecres SPA, Verona, Italy) was injected into the vertebral body or IVC area with a 3.5 mm side open bone cement injector (Shanghai Kinetic Medical Co., Ltd., Shanghai, China) until the bone cement distribution was well dispersed and satisfactory. The surgical procedures and methods of the two groups were the same and were completed by the same group of surgical staff.

### Postoperative treatment

The patients were kept in the supine position for 24 h postoperatively. The patients were still on bed rest for 1 month. Lumbar and back support was used to protect the patients from getting off the bed within 1–2 months after surgery. Routine comprehensive anti-osteoporosis treatment postoperatively was conducted by oral administration of calcium carbonate D3 600 mg/day, alfonetinol tablets 0.5 g/day, alendronate sodium tablets 10 mg/week) or intravenous infusion of zolefrononic acid (5 mg/year). X-ray films of injured vertebra were reviewed periodically after surgery.

### Efficacy evaluation index

Intraoperative dispersion morphology, the amount and leakage rate of bone cement, operation time, and the incidence of new adjacent vertebral fracture during the follow-up period were records in the two groups.

Before surgery, at the first day after surgery, the first year after surgery, and the last follow-up, VAS was used to assess the degree of lumbar and back pain of patients [12], and ODI was used to assess the degree of activity limitation of patients [13].

The anterior edge height and Cobb angle of the injured vertebral body were measured on the lateral spine X-ray image before surgery, at the first days after surgery, the first year after surgery, and the last follow-up. According to the data, the following indicators were calculated: (1) rate of vertebral compression is the height of the injured vertebral anterior edge divided by the average height of the adjacent upper and lower vertebral anterior edge [14]. (2) Correction degree of vertebral compression = (the rate of anterior vertebral compression preoperatively—the rate of anterior vertebral compression postoperatively)/the rate of anterior vertebral compression preoperatively × % [14]; (3) correction degree of kyphosis = (preoperative Cobb angle − postoperative Cobb angle)/preoperative Cobb angle × % [5].

Bone cement leakage was observed after operation. According to the anatomical characteristics of the spine, the leakage sites were classified as intraspinal leakage, anterior vertebral leakage, intervertebral disc leakage, paraspinal vein leakage, and puncture needle leakage.

### Statistical analyses

SPSS19.0 (IBM Corp., Armonk, NY, USA) statistical software was used for statistical analysis. Measurement data were expressed as mean ± standard deviation. The Levene test was used to test the homogeneity of variance. Independent sample *t* test was used for inter-group comparison. One-way ANOVA (Bonferroni or Dunnett T3) was used for comparison between groups at different time points, and repeated measurement analysis of variance was used for intragroup comparison at different time points. The count data were tested by *χ*^*2*^ test. *P* < 0.05 was considered statistically different.

## Results

The two groups were followed for at least 24 months. There were no significantly differences in the follow-up time, operation time, intraoperative blood loss, number of fluoroscopy, bone cement leakage, and the incidence of new adjacent vertebral fracture between the two groups (all *P* > 0.05). The leakage rate of bone cement in the blocky group was 16.67%, including 3 cases of anterior vertebral leakage, 1 case of intervertebral disc leakage, and 1 case of paravertebral vein leakage. The leakage rate of bone cement in the spongy group was 21.21%, including 1 case of anterior vertebral edge leakage, 3 cases of intervertebral disc leakage and 3 cases of paravertebral vein leakage. However, there were no related clinical symptoms in the both groups. The amount of bone cement injected into the blocky group was significantly higher than that of the spongy group (*P* = 0.000)(Table 2).

VAS and ODI postoperatively of the two groups were significantly reduced at all time points (all *P* = 0.000) and were maintained at the last follow-up. VAS and ODI postoperatively decreased significantly in the spongy group compared with the blocky group at all time points (all *P* = 0.000) (Table 2).

The correction degrees of kyphosis and vertebral compression postoperatively in the two groups were significantly corrected, but gradually decreased over time (*P* < 0.05), and these correction rates of the blocky group were significantly higher than these of the spongy group, and the postoperative losses over time were also more serious (loss rate of correction degree of kyphosis at last follow-up 18.25% vs*.* 10.65%; loss rate of correction degree of vertebral compression 16.34% vs. 7.35%) (Table 2).

## Discussion

Since Galiber et al. [15] applied PVP in the treatment of 1 case of C2 vertebral invasive hemangioma in 1984, PVP has gradually become one of the effective methods for the treatment of vertebral tumor and OVCFs due to its advantages of simple operation and definite efficacy [12, 16, 17]. KD is a rare special type in OVCFs. After minor trauma, vertebral collapse and kyphosis gradually appear, which are related to vertebral ischemia and necrosis and the formation of vertebral pseudarthrosis. The occurrence of KD is usually manifested as intractable lumbago and back pain, and in severe cases, nerve damage may occur [18, 19]. X-ray and CT examination shows IVC in the vertebral body, and MRI suggests limited fluid filling in the vertebral body [1, 2, 20]. IVC is mainly located in the thoracolumbar region, and most of the fractures are wedge-shaped fractures, with fractures occurring near the upper and lower endplates of the vertebral body [11, 21].

Due to the presence of IVC and pseudarthrosis, the injured vertebra of KD can flex during spinal flexion activities, which can widen the cracks in the vertebral body [22]. The partial correction of collapsed vertebral height and kyphosis can be achieved when the spine is extended. In PVP treatment, the bone cement is often confined to the diffusion in the crack, which has the effect of maintaining the extension and correcting kyphosis, without the need of PKP balloon expansion and reduction. Spontaneous reduction can occur in patients with KD in posterior extension without further balloon expansion reduction [23, 24]. Heo et al. [25] reported that excessive reduction is likely to accelerate the process of vertebral ischemia and necrosis, leading to severe recollapse, so excessive reduction of injured vertebra during surgery should be avoided. PKP has no obvious clinical advantages over PVP [25]. Therefore, we selected PVP combined with postural reduction to treat KD.

A small dose of bone cement can restore the mechanical properties of the fractured vertebral body, and the amount of bone cement injected has no obvious correlation with the analgesic effect [26, 27]. Even 1.5 ml of bone cement injected into each vertebral body can obtain satisfactory analgesic effect [26, 27]. Biomechanical studies have confirmed that vertebral strength can be restored by injecting about 2 ml bone cement, and vertebral stiffness can be restored by injecting about 4 ml bone cement [27]. In this study, both groups have reached the requirements of restoring vertebral strength and stiffness. However, the amount of bone cement injected is a one-sided index to reflect the benefit of bone cement and cannot reflect the distribution of bone cement in vertebral body. The diffusion volume of bone cement can more reasonably reflect the distribution of bone cement in vertebral body. This study suggests that different distribution patterns of bone cement are closely related to the degree of osteoporosis, the degree of compression, the position and internal morphology of IVC, and the degree of peripheral sclerosis of IVC.

In this study, VAS and ODI postoperatively were significantly reduced in both groups and were maintained to the last follow-up. Li et al. [28] found that VAS and ODI were significantly improved at the last follow-up compared with those before treatment in patients with KD after intensive vertebral treatment. In this study, spongy group is superior to the blocky group in terms of pain relief and functional recovery. Due to the obstruction of the ossification band and fibrous membrane at the edge of IVC, bone cement of the blocky group was filled in the IVC region in the form of solid mass, resulting in the failure of effectively filling of bone cement in other gaps and osteoporosis areas around the crack. The limited bone cement mass cannot be connected with the upper and lower adjacent endplates and cannot strengthen cancellous bone of vertebrae, which is more prone to stress shielding leading to recollapse [29], and cannot support the normal physiological stress from the body resulting in the continued existence of pain symptoms caused by osteoporosis [30]. In the spongy group, the bone cement was intercalated trabeculae with diffuse distribution and had high degree of vertebral strengthening. In this study, the average injected amount of bone cement per vertebra in the blocky group was 4.7 ± 1.55 ml vs. 4.0 ± 1.16 ml in the spongy group. Although the injected amount was more in the blocky group than in the spongy group, the spongy group showed better recovery of pain relief and function after treatment. Many scholars also found that the injected amount of bone cement was not proportional to the analgesic effect [26, 27]. Therefore, it is suggested that the fractured cracks should be filled and the cement should be better distributed in the vertebrae in the PVP treatment. We found that the postoperative correction degrees of vertebral compression and kyphosis in the two groups gradually decreased over time, suggesting that the postoperative vertebral height and Cobb angle gradually lost, which was consistent with previous findings [17, 31]. The loss of the blocky group was more obvious than that of the spongy group, which was related to the distribution pattern of the bone cement itself.

The correction rate of postoperative kyphosis and vertebral compression was more obvious in both groups, but these correction rates of blocky group were more obvious than that of spongy group. Course of the disease, age, preoperative Cobb angle, and vertebral compression rate of the blocky group were significantly higher than those of the spongy group, while the anterior vertebral height and BMD of the blocky group were significantly lower than those of the spongy group. It is suggested that the condition of KD in the blocky group is more serious than that of the spongy group. So the formation time of IVC is longer, the volume of IVC is larger, and the pseudarthrosis is obvious. Therefore, when the spine is in the position of posterior extension, the vertebral body has a large reduction space and presents the “open-mouth state.” The injected bone cement presents a mass distribution, which effectively restore the height of vertebral body and correct kyphosis.

The bone cement leakage rate of the blocky group was 16.67% lower than that of the sponge group (21.21%), but there was no statistical difference between the two groups. Krauss et al. [32] reported that the bone cement leakage rate was 18.2% in the PVP treatment of KD. Wang et al. [33] reported that the bone cement leakage rate was 7.4% in the PKP treatment of KD. In the blocky group, the anterior vertebral edge leakage was the main part, while in the spongy group, the intervertebral disc leakage and the paravertebral vein leakage were the main part. However, there were no related clinical symptoms in both groups. In the blocky group, due to the obstruction of the sclerosis band and fibrous membrane around the IVC area, the injection of bone cement showed a lumped distribution. The bone cortex in the anterior edge of the vertebral body was often incomplete, which could leak out along the anterior edge to the front of the vertebral body. Therefore, the incidence of leakage in the anterior edge of the vertebral body of the blocky group was the highest. However, the spongy group of bone cement was widely inserted into the trabecular space and distributed in a diffuse manner, which was easy to be squeezed into the intervertebral disc and the paravertebral vein plexus. In order to prevent the occurrence of bone cement leakage, the operator should carefully analyze the imaging data before treatment, fully understand the IVC position, size and fracture site, and grasp the position of pedicle injection.

The blocky group had an incidence of adjacent vertebral fracture of 16.67% vs. the spongy group of 9.09%. But there was no statistical difference between the two groups. It indicates that the blocky distribution of bone cement is more likely to lead to the occurrence of adjacent vertebral fractures. Yang et al. [34] found that 14.1–39.1% OVCFs patients in the PVP treatment experienced an adjacent vertebral fractures during the first year after surgery. In the blocky group, the vertebral body was seriously compressed and kyphosis was obvious. The biomechanics of adjacent segments changed greatly. The mechanical transfer between the vertebral body and the intervertebral disc was changed, resulting in the increase of the stress of adjacent vertebrae and the higher probability of new refracture. In addition, the bone cement was filled in the IVC area in the form of solid mass, which was more prone to stress shielding, increased the stress load of adjacent intervertebral discs and vertebral bodies, and increased the risk of recurrent fractures [29]. The bone cement in the spongy group was evenly distributed, which made the stress of the whole vertebral body relatively evenly distributed, and reduced the concentrated stress on the adjacent vertebral body and intervertebral disc. Therefore, the complication rate of new adjacent vertebral fracture is low.

This study has some limitations. Firstly, KD is relatively rare and easy to be complicated with OVCFs and multiple fractures, so there are few cases to meet the inclusion criteria. Therefore, the number of cases in this study was small, and the sample size needs to be expanded for further verification and analysis. Preoperative ICV morphology and size were not measured in three dimensions. Biomechanical studies of cement distribution in the vertebrae on adjacent vertebral bodies and intervertebral discs were lacking to support the results. The researchers were not completely blinded in the collection of data, which may lead to bias in the data. Therefore, the current findings provide a clinical reference, but need to be validated in further multi-center, randomized, double-blind clinical trials.

## Conclusions

PVP can effectively relieve pain, improve function, restore vertebral height, and correct kyphosis in the treatment of KD. Compared with the spongy group, the blocky group had longer course, older age, and more serious kyphosis, vertebral compression, and osteoporosis. The amount of bone cement injected into the blocky group was significantly higher than that of the spongy group. Furthermore, the correction degrees of kyphosis and vertebral compression in the blocky group were more obvious than these in the spongy group, but the postoperative losses were also more serious. However, VAS and ODI were significantly lower in the spongy group than in the blocky group. Therefore, the spongy distribution pattern should be formed during the injection of bone cement to obtain better therapeutic effect.

## Data Availability

The datasets used and/or analyzed during the current study are available from the corresponding author on reasonable request.

## Abbreviations

PVP: Percutaneous vertebroplasty
PKP: Percutaneous kyphoplasty
OVCFs: Osteoporotic vertebral compression fractures
IVC: Intravertebral vacuum cleft
ODI: Oswestry Disability Index
VAS: Visual analog scale
IVP: Intravertebral vacuum phenomenon
BMD: Bone mineral density
